# Supplementary material for: Innovating within or outside dominant food systems? Different challenges for contrasting crop diversification strategies in Europe
Source: PLoS One. 2020 Mar 12;15(3):e0229910. doi: 10.1371/journal.pone.0229910 (PMC7067481; doi:10.1371/journal.pone.0229910)
Supplement: S1 Appendix — (DOCX) [file pone.0229910.s001.docx]

**Supporting information**

**S1 Appendix: guidelines and structured framework to support innovation teams in identifying barriers to crop diversification in the DiverIMPACTS project**

***Starting identifying barriers to diversification before the second round of co-innovation***

A range of barriers to crop diversification exist at all levels of the value chain: farms, collectors, processors, retailers, consumers. These limiting factors may come from the past evolution of agrifood systems and altogether, they can create a situation of “lock-in”, which means that innovations based on diversification are difficult or impossible to implement although they may have more positive outcomes than the existing practices.


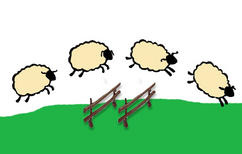
Experts and scientists of Work Package 5 (WP5) will support innovation teams in developing technical and organisational solutions to overcome them: at the farm level (5.2), at the logistic, processing and retail level (5.3), designing adapted contracts between actors (5.4) and investigating whether and how consumers can value products coming from diversification (5.5).

Overcoming barriers

Before starting this work, it is crucial to get an initial comprehensive view of the barriers encountered across the 25 cases.

*Simple things to do before the next round of co-innovation workshops*

In the first round of workshops, you have started identifying some causes (causal analysis) that may be barriers to diversification. Before the second round of workshops, you will meet some relevant stakeholders of your case to help you define better your objectives. This is a perfect occasion to get more elements about the barriers to diversification from other perspectives!

In the table below, you will find some key elements to keep in mind when you will talk with stakeholders before the next round of workshops.

**Elements to keep in mind during interviews with actors/stakeholders**

| **Level of barriers** | **Points to keep in mind when talking with actors** | **Some barriers to diversification you should be aware of (among other)** |
| --- | --- | --- |
| **Farm level** | - Why would farmers be reluctant to the diversification you propose? - Why would farmers be enthusiastic about the diversification you propose? - What are their main issues and priorities? - Can you see common points among the farmers who are willing to diversify? - Can you see common points among the farmers who are not willing to diversify (age, size, ecological context, area, values and objectives, type of farms, level of infrastructure and equipment etc.)? | Availability of inputs (seeds, adapted phytosanitary products) |
|  |  | Availability of machinery (sowing, harvesting, screening…) |
|  |  | Availability of knowledge/advice/references/skills/technics |
|  |  | Economic profitability (yields, costs, prices, subsidies) |
|  |  | Social pressure (being a "good/normal" farmer) |
|  |  | Existing infrastructure, equipment and investment for cultivation/storage/processing |
|  |  | Fear of uncertainty, complexity, risks (on yields and/or prices)  Competition with other crops for land or labour force |
| **Logistic, processing and retail** | - Why would the different actors of the downstream value chain (collectors, processors, retailers) be reluctant to the diversification you propose? - Why would they be enthusiastic about the diversification you propose? - What are their main issues and priorities? - Do the new products compete with existing products? | ***Collecting and storage*** |
|  |  | No equipment to screen the products (if various products harvested together) |
|  |  | Missing or inadequate transport means |
|  |  | Transport too complicated/expensive for small/variable/scattered quantities |
|  |  | Inadequate, insufficient or not flexible storage capacities |
|  |  | ***Transformation and processing*** |
|  |  | Competition with other sources of raw materials, processing chains not adapted to small quantities |
|  |  | Variability in terms of quantity and quality of the raw material |
|  |  | Lack of knowledge and adapted methods/technologies to process the products |
|  |  | ***Distribution*** |
|  |  | Variability in terms of quantity and quality of the processed product |
|  |  | Retailers not willing to sell the products because no market (see next points). |
| **Contract arrangements** | - Which types of arrangements exist between the different actors of the value chain (farmers, collectors, processors, retailers)? - In which extent are these arrangements favourable or unfavourable to diversification? Why? - Would these arrangements be suitable for the products coming from diversification or should new arrangements be designed? - What are the main challenges in terms of traceability, quality, quantity, sharing of the added value between the actors? | Lack of long-term and formalized coordination between the actors of the value chain |
|  |  | No ensured minimal and stable supply of products along the value chain |
|  |  | No long term specifications about the quality and traceability of products |
|  |  | Added value is not fairly shared along the value chain |
|  |  | Lack of flexibility in arrangements between actors to adjust to hazards and changes |
| **Consumer level** | - Is there a market to sell the products? Which markets? - In which extent can consumers be aware of the fact that the product they buy comes from diversification? - Would they be ready to value/prefer such products and pay more for them? | No/limited existing market for the products |
|  |  | Benefits of diversification not visible or not valued by consumers |

*"products" stand for "products coming from diversification" (directly, or indirectly e.g. animal fed with new crops)*

The stakeholders you will meet before the next round of workshops and the way you will interact with them or interview them may be different for each case. The table offers some guidelines and topics that may be discussed with your stakeholders to get a picture as wide as possible of the different barriers your case will face.

You are free to use it the way you think to be more relevant and adapted. You know better your context! You could for example read this table each time you go to an interview to have in mind relevant topics to discuss or print it and ask more systematically the questions to stakeholders.

After interviews, do not forget to keep track of what was said (this is a precious material). In the second round of workshops, each case team will have a 30 minutes interview with scientists from WP5. This table will be the basis of this interview. You will go through the different topics based on the information you have collected. To prepare this interview, you may write or draw before the workshop a short synthesis about what your different stakeholders said and your own perception of the barriers to diversification at the different levels. To help you, you can use the template provided below but you are free to make it a different way. The crucial point is to present a global view of barriers at different levels based on the perspective of your stakeholders
