# Supplementary figures and images for: Innovating within or outside dominant food systems? Different challenges for contrasting crop diversification strategies in Europe
Source: PLoS One. 2020 Mar 12;15(3):e0229910. doi: 10.1371/journal.pone.0229910 (PMC7067481; doi:10.1371/journal.pone.0229910)

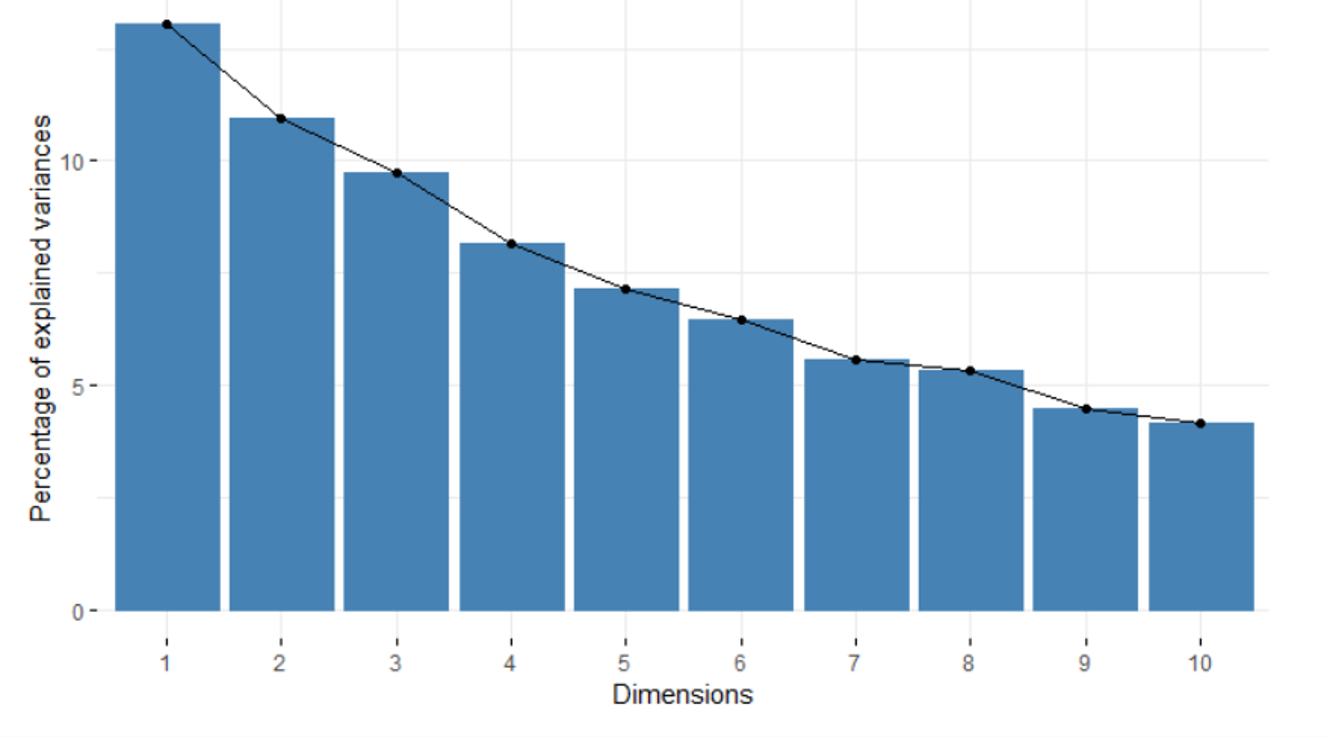

Supplement: S1 Fig — Cumulated, dimensions 1 to 4 explain 42% of variance. (TIF) [file pone.0229910.s002.tif]

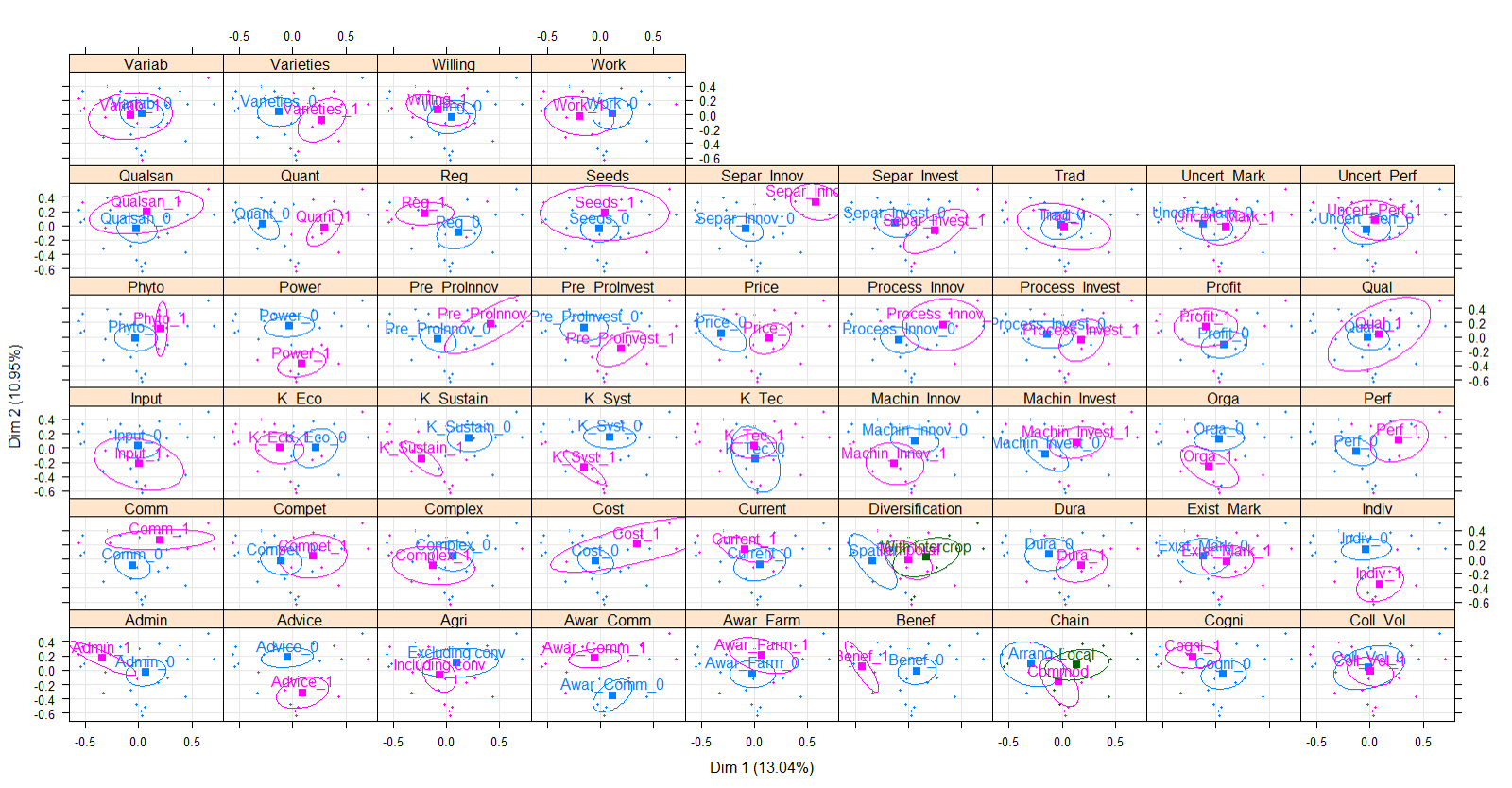

Supplement: S2 Fig — Ellipses around the different modalities of each variable indicate significant difference with a confidence of 95%. Codes for barriers are presented in S1 Table and for supplementary variables in Table 1. (TIF) [file pone.0229910.s003.tif]

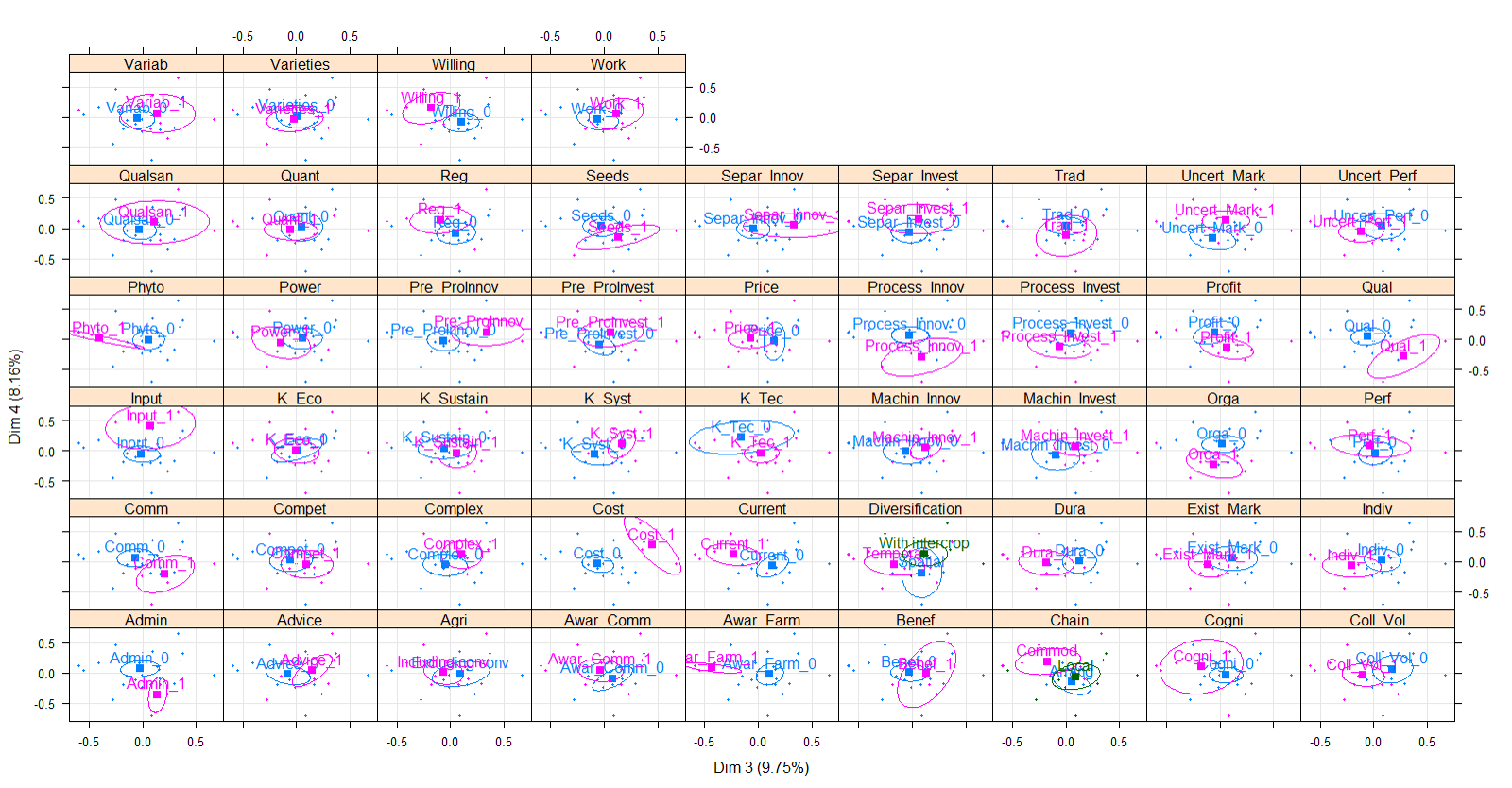

Supplement: S3 Fig — For legend, see S2 Fig. (TIF) [file pone.0229910.s004.tif]
