# Supplementary material for: Innovating within or outside dominant food systems? Different challenges for contrasting crop diversification strategies in Europe
Source: PLoS One. 2020 Mar 12;15(3):e0229910. doi: 10.1371/journal.pone.0229910 (PMC7067481; doi:10.1371/journal.pone.0229910)
Supplement: S1 Table — (DOCX) [file pone.0229910.s005.docx]

**S1 Table. V-test of coordinates of supplementary variables on dimensions 1 to 4 in the MCA of barriers to crop diversification.**

|  | **Dim 1** | **Dim 2** | **Dim 3** | **Dim 4** |
| --- | --- | --- | --- | --- |
| **Spatial** | -2,27 | -0,23 | 0,7 | -1,56 |
| **Temporal** | -0,02 | -0,14 | -2,1 | -0,56 |
| **With intercrop** | 1,87 | 0,33 | 1,53 | 1,84 |
| **Arrang** | -1,95 | 0,62 | 0,4 | -1,31 |
| **Commod** | -0,26 | -1,66 | -1,82 | 2,21 |
| **Local** | 1,8 | 1,05 | 1,38 | -1,02 |
| **Only organic** | 1,11 | 1,24 | 1,23 | -0,25 |
| **Including conv** | -1,11 | -1,24 | -1,23 | 0,25 |

For each 2-axes projection, only the supplementary variables the most linked to the axes were kept in the analysis (cut-off: v-test absolute value higher than 0.8 for one of the dimension, corresponding to an alpha error of 10%).
