# Supplementary material for: Innovating within or outside dominant food systems? Different challenges for contrasting crop diversification strategies in Europe
Source: PLoS One. 2020 Mar 12;15(3):e0229910. doi: 10.1371/journal.pone.0229910 (PMC7067481; doi:10.1371/journal.pone.0229910)
Supplement: Attachment — Submitted filename: Response_to_reviewers.docx [file pone.0229910.s007.docx]

Dear editors and reviewers,

We thank you for your positive feedbacks on the changes we made based on your initial comments. Please find below, the new changes we made to address your “second round” of comments (in italic).

*Please consider all relevant language issues raised by the two reviewers. It is recommended to consult a professional native speaker for general language editing to further improve the clarity of your manuscript.*

The whole manuscript has been proofread and edited by a native professional English translator.


*Reviewer #1: The authors have well responded to the comments. Still, a thorough language check is needed. The text is comprehensible but there are still so many incidences where the text is not clear enough, the used terms probably not always the correct ones or where grammar issues cause some confusion (e.g. prepositions, the use of plural/singular in composed terms, commas, using “the”, and the s in the present tense). In the following some detailed comments – but I stopped with marking language issues after some lines.*

The whole manuscript has been proofread and edited by a native professional English translator.

*L26-28 instead of always writing “a” type of, use “the” type of*

We have replaced “the type” by “a type” along the manuscript.

*L32 where crop diversification increases (with s)*

We have added a “s”.

*L43: The transition … requires (with s)*

We have added a “s”.

*L73: of farmers taking the related risks (not whatsoever risks)*

We have added “the related risks”.

*L82: approaches (with s)*

We have added a “s”.

*L89-92: complicated sentence, check with a native*

The sentence was edited by a native.

*L101-103: be more specific, check the correct wording. Why is “reduced inputs” a sustainability benefit? Did you mean something like relying less on external inputs? “yields gaps” – closing yield gaps? I think the s from risks is not needed here. Normally we use the term ecosystem services – did you mean this by ecosystemic services, or is that something else?*

Wording was checked by a native. We added “reduced” yield gaps to be more specific and “ecosystem services” instead of “ecosystemic services”. “Reduced inputs” was replaced by “reduced dependency on external inputs” to be clearer.

*L103: have proven*

We have replaced it by “have shown”.

*L104: of “the” food systems
….am more or less stopping here with my specific language, grammar etc notes…*

The native proof-reader judged that “barriers at all levels of food systems” was ok. The whole text was edited by this native proof-reader.

*L119: lens? Is that lentils?*

We meant lentils and changed the wording accordingly.

*Tab 1 (and Tab2): in the first row of Tab 1, better use Nb as used in Tab 2, good for consistency, instead of number. Even though the legend explains it, number may cause people think that you talk about the number of cases, such as 3 cases for the first row, 8 cases in the second and so forth – avoid such potential confusion.
..if ever in Tab 2 you mean Nb as case ID. Am finally not sure what you mean by “occurrence of barriers among the 25 case-studies”, row one: it occurs in case 21 or it occurred 21 times? If you mean 21 times then better use “n”. If you mean case 21 then better use something like ID.*

In Tab 1, the number is the case ID, so we changed “Case number” by “Case ID”. In the legend of Tab 1, we now specify “The cases are called by their ID in the DiverIMPACTS project, to make it easier to find more information on the project website: <https://www.diverimpacts.net/case-studies.html>””

In Tab 2, the number relates to the number of times the barrier occured, so as suggested we use now « n” and in the legend indicate: “number of occurrences of the related barrier among the 25 case-studies”.

This clarification was indeed very useful!

*L372 what are “contrasted” positive impacts? May be you meant complementary or cumulative? Or simply positive impacts is enough?*

Positive impacts is enough and we deleted “contrasted”.

*L382 the use of “external” inputs? Cycling of internal goods is quite useful I think, you probably mean less dependency on inputs to be bought.*

We replaced it by “could reduce the dependency on external inputs”.

Reviewer #3:

*217 : I suggest removing the 5 in front of « different innovation teams »*

Here we wanted to explain that we organised 5 different workshops and that each workshop involved 5 innovation teams. So we modified the sentence to be clearer: *“In a first round, 5 workshops were organised, each involving 5 different innovation teams out of 25. “*

*696-698 : there are two times the same verb « are growing » in the sentence*

We corrected that and the native editor reformulated the sentence as such: “Industrialised countries are witnessing the growth of intermediate food value chains”

707-708 : to be rephrased « hybrid configurations result in solving OF creating more barriers »

We rephrased it as such: ““hybrid configurations remove or create more barriers”.
